# Cecal CircRNAs Are Associated With the Response to *Salmonella Enterica* Serovar Enteritidis Inoculation in the Chicken

**DOI:** 10.3389/fimmu.2019.01186

**Published:** 2019-05-31

**Authors:** Linna Zheng, Liying Liu, Lili Lin, Hui Tang, Xinzhong Fan, Hai Lin, Xianyao Li

**Affiliations:** ^1^Shandong Provincial Key Laboratory of Animal Biotechnology and Disease Control and Prevention, College of Animal Science and Technology, Shandong Agricultural University, Tai'an, China; ^2^College of Life Sciences, Shandong Agricultural University, Tai'an, China

**Keywords:** chicken, circular RNA, *Salmonella enterica* serovar Enteritidis, expression profile, metabolism, immunology

## Abstract

Circular RNAs (circRNAs) are a class of endogenous noncoding RNA, which is different from linear RNA. CircRNA is an RNA molecule with a closed loop structure formed by reverse splicing. CircRNAs have been studied in several organisms, however, the circRNAs associated with the response to *Salmonella enterica* serovar Enteritidis (SE) inoculation in chickens are still unclear. In the current study, Jining Bairi chickens were inoculated with SE. CircRNAs involved in the response to SE inoculation were identified through next-generation sequencing. Our results showed that there were 5,118 circRNAs identified in the control and treated groups. There were 62 circRNAs significantly differentially expressed following SE inoculation. Functional classification revealed that those significantly differentially expressed circRNAs were associated with immune system process, rhythmic process and signaling following SE inoculation. CircRNAs NC_006091.4: 65510578|65515090, NC_006099.4: 16132825|16236906, and NC_006099.4: 15993284|16006290 play important roles in the response to SE inoculation. The findings in the current study provide evidence that circRNA alterations are involved in the response to SE inoculation in the chicken.

## Introduction

*Salmonella enterica* serovar Enteritidis (SE) is one of the most common serotypes of the Salmonella bacteria reported worldwide and is the primary source of human intestinal infection (1). From the total confirmed Salmonella infections, 18% were caused by SE, and the incidence was 2.83 per 100,000 people in the United States (2). Egg-related salmonellosis costs $44 million per year in Australia (3). SE has a close relationship with the chicken, as poultry meat and egg are regarded as the main source of human foodborne infection (4). SE infection is mainly caused by oral intake of contaminated feed or water. SE can enter the bloodstream and colonize the internal organs. The cecum is the main site of Salmonella colonization (5). Many studies showed that genetic selection is an efficient way to control Salmonella infection in the chicken (6).

Circular RNAs (CircRNAs), a novel type of noncoding RNAs, compose a class of RNA developing covalently closed loop structures without 5′-3′ polarities (7) but with widespread, abundant, and tissue-specific expression across animals (8). To date, circRNA can be divided into three types, namely, intronic circRNA, exonic circRNA and exon-intron circRNA (9–11). Many circRNAs comprise one to several exons of protein-coding genes (12). CircRNA production may occur posttranscriptionally (13). Several functions have been identified for circRNAs, including transcription regulation, RNA transport (14), protein binding (15), and regulation of translation (9). CircRNAs were identified as efficient microRNA (miRNA) sponges (16, 17). Moreover, circRNAs have been found to be associated with diseases, including Alzheimer's disease, colorectal and ovarian cancer, idiopathic lung fibrosis and hepatocellular carcinoma in humans (12, 18, 19). Recent studies indicated that circular RNA expression alterations were enriched and stable in exosomes and could be a promising biomarker for cancer diagnosis (20, 21). It has been reported that circRNA alterations are involved in resistance to ALV-J-induced tumor formation in the chicken (22).

The technological breakthroughs in high-throughput deep sequencing and functional genome promote the study of circRNAs (23). A large number of circRNAs has been identified in Archaea, mice, and humans (10, 24). However, the identification of circRNAs related to certain traits in chickens is limited. In the current study, next-generation sequencing was used to detect circRNAs involved in the response to SE inoculation in the chicken. The study will lay the foundation in which circRNAs may be used as molecular markers related to the host response to SE inoculation in chickens.

## Materials and Methods

### Animals and SE Inoculation

The Jining Bairi Chicken, a China local chicken breed, used in the current study was provided by Shandong Bairi Chicken Breeding Co., Ltd (Jining, Shandong, China). The SE strain (CVCC3377) used for the inoculation was purchased from China Veterinary Culture Collection Center (http://cvcc.ivdc.org.cn/). To make the inoculant, SE were enriched in LB broth at 37°C for 16 h, pelleted at 4,000 rpm for 5 min, and diluted with sterilized PBS to an adjusted OD value of 1. The concentration of SE in the inoculant was measured by the plating method. The experimental design of animal inoculation was described in detail previously (25). In brief, 168 2-day-old SE negative Jining Bairi chickens were randomly divided into 2 groups, 84 chickens in each group. Chickens in one group were orally inoculated with 0.3 mL inoculants of 10^9^ cfu/ml as the treated (T) group, and chickens in another group were mock inoculated with the same amount of sterile PBS as the control (C) group. Twelve chickens from each of the T group and C group were euthanized by cervical dislocation for sample collection at 1, 3, 7, 14, 21, 28, and 35 days postinoculation (dpi). The cecum samples were frozen in liquid nitrogen and stored at −80°C until further RNA isolation. Samples collected at 7 dpi were selected for the current study based on the bacterial number in cecal content in the treated group. All animal procedures were approved by the Shandong Agricultural University Animal Care and Use Committee.

### RNA Extraction and circRNA Sequencing

Four samples from each T and C groups at 7 dpi were randomly selected for RNA isolation. Total RNA was isolated from each sample using the TRIzol reagent (Invitrogen, Grand Island, NY) according to the manufacturer's instructions. The concentration of RNA sample was measured using a DS-11 Spectrophotometer (DeNovix, Wilmington, DE, USA). The integrity of the RNA sample was assessed by agarose gel electrophoresis. Three qualified RNA samples were selected in each group and encoded as C1, C2, C3 and T1, T2, T3 to construct the library. In total, six libraries were constructed. Subsequently, the RNA libraries were sequenced by Illumina HiSeq2500 platform according to the manufacturer's instructions at BioMarker Technologies (Beijing, China).

### Data Analysis

Raw data were first processed using a custom Perl script. Clean reads were obtained after removing the adaptors, poly-N reads, and low quality reads and used for the downstream analysis. The clean reads were mapped to the chicken genome sequence (galGal 5.0) using the TopHat2 version 2.0.10 (26) and bowtie2 software (27). CircRNAs were predicted using CIRI (CircRNA Identifier) (28). Annotation of the circRNA was performed based on the following databases: Nr (NCBI nonredundant protein sequences), Pfam (protein family), KOG/COG (Cluster of Orthologous Groups of proteins), and Swiss-Prot (http://www.ebi.ac.uk/swissprot/). All the data have been deposited into the SRA database with an accession number of SRP158084.

### Function Analysis and Identification of Differentially Expressed circRNAs

The raw junction reads for all the samples were normalized to the number of total mapped reads and log2 transformed. The significantly differentially expressed (SDE) circRNAs between the T and C groups were identified using the DESeq 2 (29). The resulting *P*-values were adjusted using the Benjamini and Hochberg's approach for controlling the false discovery rate. Fold change > 2.0 and *P* < 0.05 were considered significant.

Gene Ontology (GO) and Kyoto encyclopedia of genes and genomes (KEGG) biological pathway enrichment analysis for SDE circRNAs were performed using the KOBAS 3.0 software (http://kobas.cbi.pku.edu.cn/index.php) (30).

### Correlation Between circRNAs, miRNAs, and Genes

miRNAs mediated by SDE circRNAs were predicted using miRanda (31). The number of miRNAs interacting with each circRNA and the number of circRNAs interacting with each miRNA were counted. The genes targeted by circRNA-mediated miRNAs were mapped to the chicken functional interaction network in the Reactome database using the Reactome FI network plugin in the Cytoscape 3.5.1 software (32).

### Validation of circRNAs Expression Through qRT-PCR (Quantitative Real-Time PCR)

The RNA samples used for quantitative real-time PCR (qRT-PCR) validation were the same as those used for sequencing. A total amount of 10 μg RNA per sample were digested with 20 U RNase R (Epicenter, Chicago, IL) at 37°C for 1 h and purified with phenol/chloroform/isoamyl alcohol. One microgram of digested RNA was reverse transcribed into cDNA using a PrimeScriptTM RT reagent Kit with genomic DNA Eraser (Takara, Dalian, China) according to the manufacturer's instructions. The qRT-PCR was performed using the 7500 Fast Real-Time PCR System (Applied Biosystems, Foster City, CA) with a PCR mixture (20 μL) containing 10 μL SYBR Green qPCR Mix (2x), 0.5 μL (0.2 μM) forward primer, 0.5 μL (0.2 μM) reverse primer, 2 μL cDNA, and 7 μL ddH_2_O. The amplification conditions were as follows: 1 cycle of 95°C for 10 min, followed by 40 cycles of 95°C for 5 s, 54°C for 15 s. GAPDH was used as the internal standard. All reactions were performed in triplicate. The sequences of specific outward-facing primers used in the qRT-PCR are listed in Table 1 and were synthesized by Sangon Biotech (Shanghai, China). The relative expression of the validated circRNA was analyzed using the 2^−ΔΔ*CT*^ method (33). The data were represented as the mean ± standard deviation. The student's *t*-test was used to assess the difference in expression of each validated circRNA between the two groups. *P* < 0.05 was considered significant.

**Table 1:** Sequence of primers used for qRT-PCR validation.

**Gene name**	**Primers (5^′^ → 3^′^)**
NC_006113.4:2670958|2679178	CCCAGGATTCATGAGACACC
	AGAGATCTGCCAGGGCTGTA
NC_006096.4:10814512|10838667	CTCGCACTGTTCACCTTTCA
	ACCTGTGAGACCAGCAGCTT
NC_006115.4:3194855|3195107	ACCTCCTTCCCCCTTAGGTT
	CACGTAGATGTCCACGGGTA
GAPDH	CTACACACGGACACTTCAAG
	ACAAACATGGGGGCATCAG

## Results

### Data Quality and circRNAs Identification

In total, 83.37 Gb clean data were obtained from the 6 samples. The percentage of bases with Q30 was 90.95%, 90.42%, 90.49% and 90.05%, 90.39%, 90.12% in C1, C2, C3 and T1, T2, T3 groups, respectively. The average number of clean reads in the C group and T group was 97,780,662 and 91,181,675, respectively. The clean reads from each sample were aligned with the chicken reference genome (galGal5.0); the rate of mapped reads was 98.86–99.66% (Table 2).

**Table 2:** Information of the clean and mapped data.

**Sample**	**Number of Clean reads**	**Average number of Clean reads**	**Number of mapped reads**	**Average number of mapped reads**
C1	90,530,234	97,780,662	89,644,352 (99.02%)	96,836,969
C2	110,491,612		109,235,962 (98.86%)	
C3	92,320,140		91,630,592 (99.25%)	
T1	93,383,800	91,181,675	93,063,694 (99.66%)	90,707,709
T2	85,106,242		84,529,812 (99.32%)	
T3	95,054,982		94,529,620 (99.45%)	

*C1, sample 1 in the control group*.

**Figure 1:**
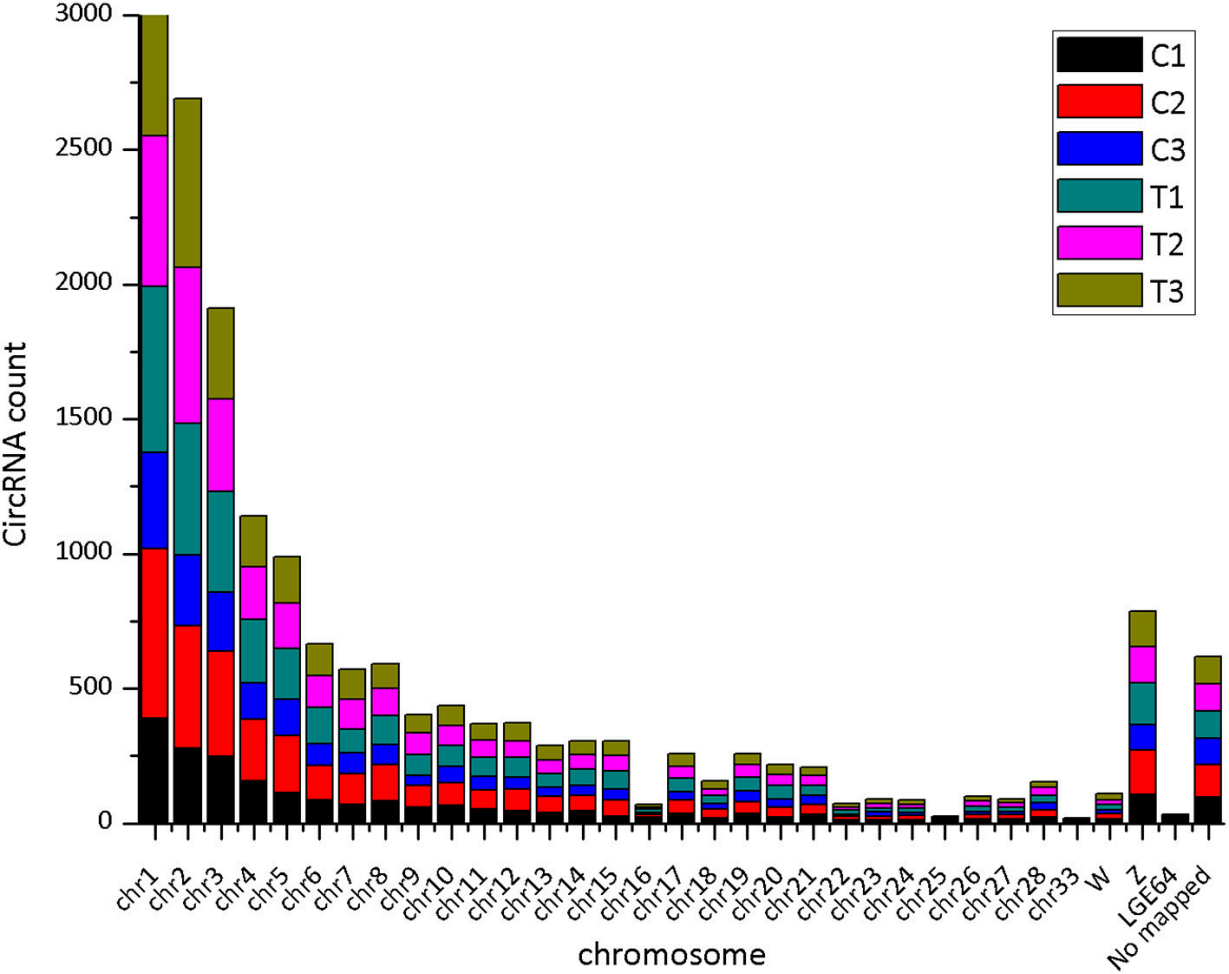
The number of expressed circRNAs on each chromosome.

The CIRI software was used to predict circRNAs. There were 5,118 circRNAs identified across the six samples. The number of circRNAs in C1, C2, C3, T1, T2, and T3 was 2,239, 3,418, 2,091, 3,264, 2,989, and 2,893, respectively. The distribution of identified circRNAs on the chromosomes was not even. There were 96.7% circRNAs mapped on Chr1-28, Chr33, ChrW, ChrZ, and LGF64. The accumulative number of circRNAs across the six samples was more than 1,000 on the Chr1-4, 500-1,000 in Chr5-8 and ChrZ, 100-500 in Chr9-15, Chr17-21, Chr26, Chr28, and ChrW, 50-100 in Chr16, Chr22-24, and Chr27, and less than 50 in Chr25, Chr33, and ChrLGE64 (Figure 1). CircRNAs were mainly located in the exon, intergenic, and intron regions. There were 86.8% circRNAs mapped to the exon region (Table 3). The coverage of circRNAs (average length of the circRNA × number of circRNAs/the length of the chromosome) on each chromosome ranged from 0.003 to 1.479. The coverage of circRNAs on Chr16 was the largest (1.479). The coverage of circRNAs was >0.1 on Chr17 and ChrLEG64, < 0.01 on Chr25 and Chr33 (Supplementary File 1).

**Table 3:** Distribution of circRNA parental genes.

**Type**	**C1**	**C2**	**C3**	**T1**	**T2**	**T3**	**Sample average**
Exon	80.573	88.849	86.476	87.784	88.410	88.457	86.758
Intergenic	14.208	6.145	8.206	7.567	6.492	7.146	8.294
Intron	5.219	5.005	5.318	4.648	5.099	4.397	4.948

### Differentially Expressed circRNAs Responding to SE Inoculation

**Figure 2:**
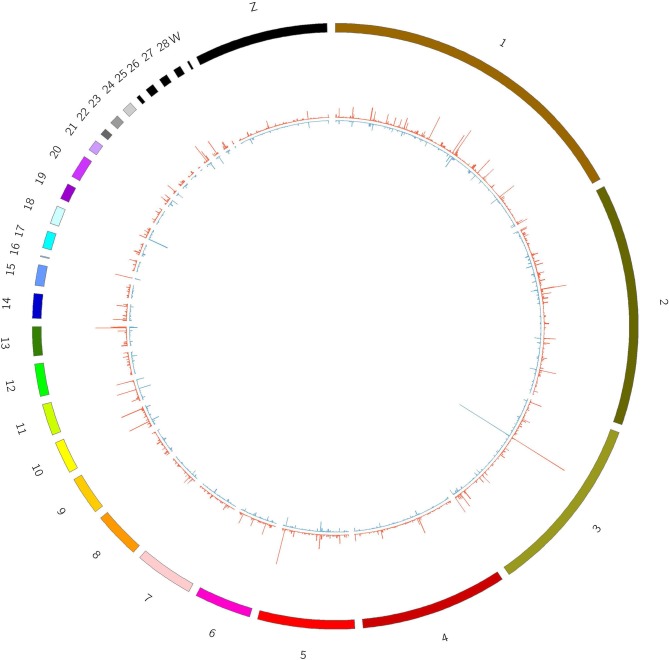
Expression of circRNAs in T and C groups. Outline corresponds to the reference genome. The inside corresponds to the average chromosome coverage across the C and T samples. Blue = C group, red = T group.

The junction reads in each sample were counted as the expression level of circRNAs. The expression of circRNAs varied across different regions within each chromosome and was different between the T and C groups (Figure 2).

The SDE circRNAs between the treated and control groups were identified through the DESeq2 software. There were 62 SDE circRNAs between the two groups including 30 upregulated and 32 downregulated circRNAs (*P* < 0.05, fold change >2). There were more than 5 SDE circRNAs located on Chr1, 2, 3, and 4. There was one SDE circRNA located on Chr8, 14, 16, 17, 20, 24, 28 and W (Supplementary File 2).

**Figure 3:**
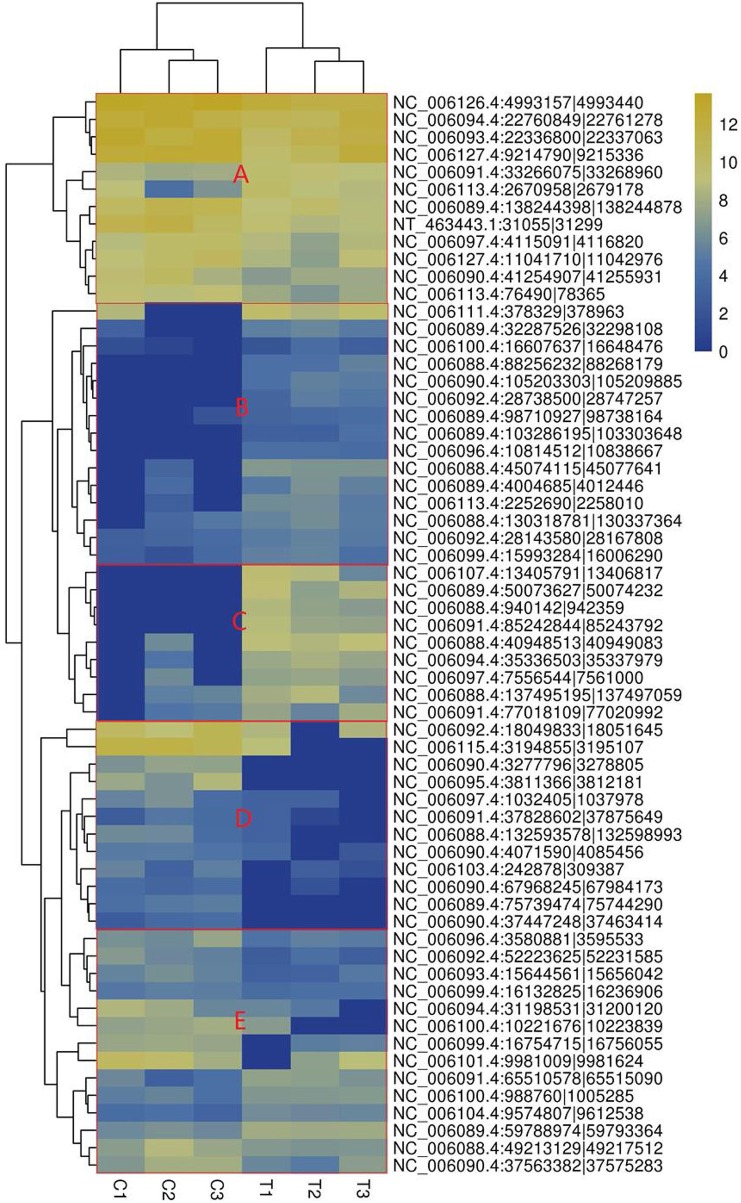
Heatmap of significantly differentially expressed circRNAs. **(A)** circRNAs with high expression in both the treated and control groups, **(B)** circRNAs highly expressed in the treated group with small difference, **(C)** circRNAs highly expressed in the treated group with great difference, **(D)** circRNAs with lower expression in the treated group. The columns represent different samples. The rows represent different circRNAs. The colors represent the level of expression of the circRNA in the sample (log2TPM). Yellow indicates higher circRNA expression level and blue shows lower circRNA expression level. T1, T2, and T3 are the samples in the T group; C1, C2, and C3 are the samples in the C group.

The heatmap based on the expression of SDE circRNAs across the six samples showed that all the SDE circRNAs were clustered into 4 groups. Group 1 composed of upregulated circRNAs in the treated group including subgroups B and C, Group 2 composed of downregulated circRNAs in the treated group (subgroup D). Group 3 composed of circRNAs with low expression in both the treated and control groups (subgroup E). Group 4 composed of circRNAs with high expression in both the treated and control groups (subgroup A) (Figure 3).

### COG Function Classification of Parental Genes

**Figure 4:**
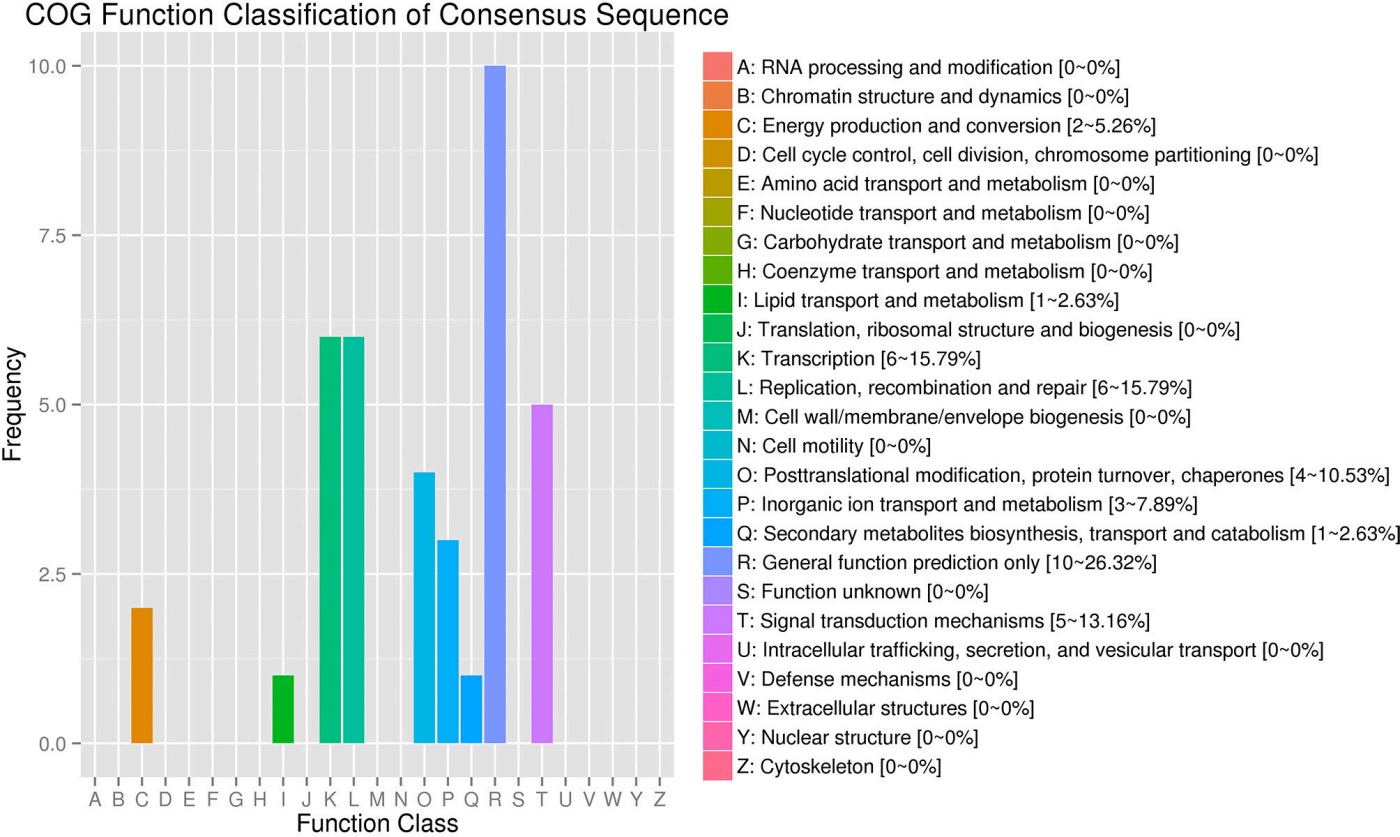
COG annotation of circRNA parental genes. The X-axis shows the COG function classification of the consensus sequence. The Y-axis shows the percentage of genes in each functional class.

The COG (Clusters of Orthologous Groups) function classification showed that the SDE circRNAs were associated with five categories: general function prediction only (R), transcription (K), replication, recombination and repair (L), signal transduction mechanisms (T), and posttranslational modification, protein turnover, chaperoned (O) with proportions of 26.32, 15.79, 15.79, 13.16, and 10.53%, respectively (Figure 4).

### GO and KEGG Pathway Analysis of Parental Genes

**Figure 5:**
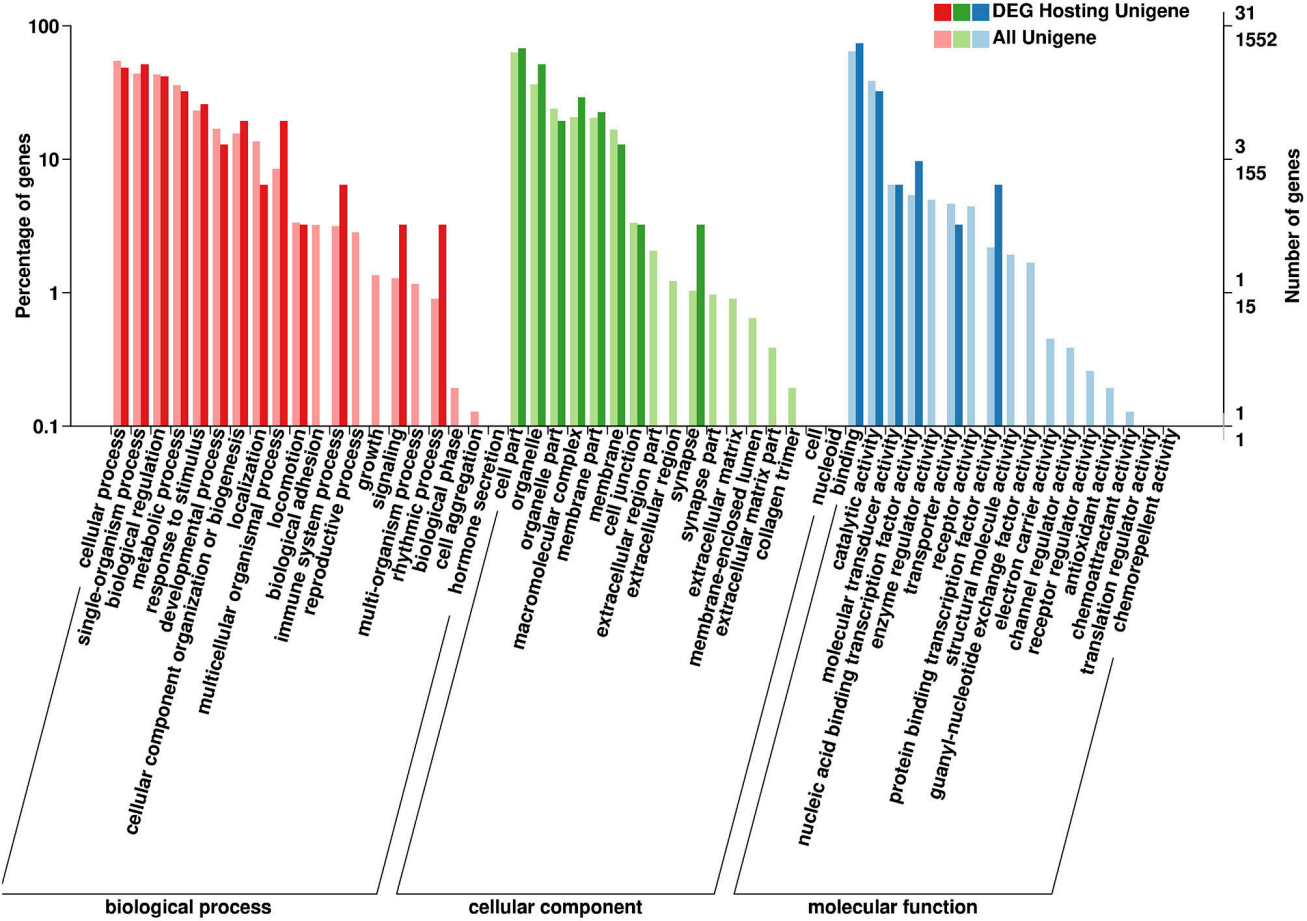
Gene Ontology (GO) annotation of circRNA parental genes. The X-axis shows the GO function classification. The Y-axis shows the percentage of genes **(Left)** and the number of genes **(Right)**.

The parental genes of the SDE circRNAs were predicted. The GO and KEGG pathway analyses were performed for those parental genes. The results of BP (biological processes), MF (molecular functions), and CC (cellular components) were shown in Figure 5. For the GO-BP, the SDE circRNAs were associated with localization, biological adhesion, immune system process, reproductive process, growth, signaling, multi-organism process, rhythmic process, biological phase, and cell aggregation. In terms of the GO-CC, the SDE circRNAs were mainly located in the synapse, organelle and the macromolecular complex. For the GO-MF, the SDE circRNAs were associated with nucleic acid-binding transcription factor activity and protein-binding transcription factor activity (Figure 5). The CLOCK gene was the parental gene of circRNA NC_006091.4: 65510578|65515090 and was involved in the rhythmic process.

**Figure 6:**
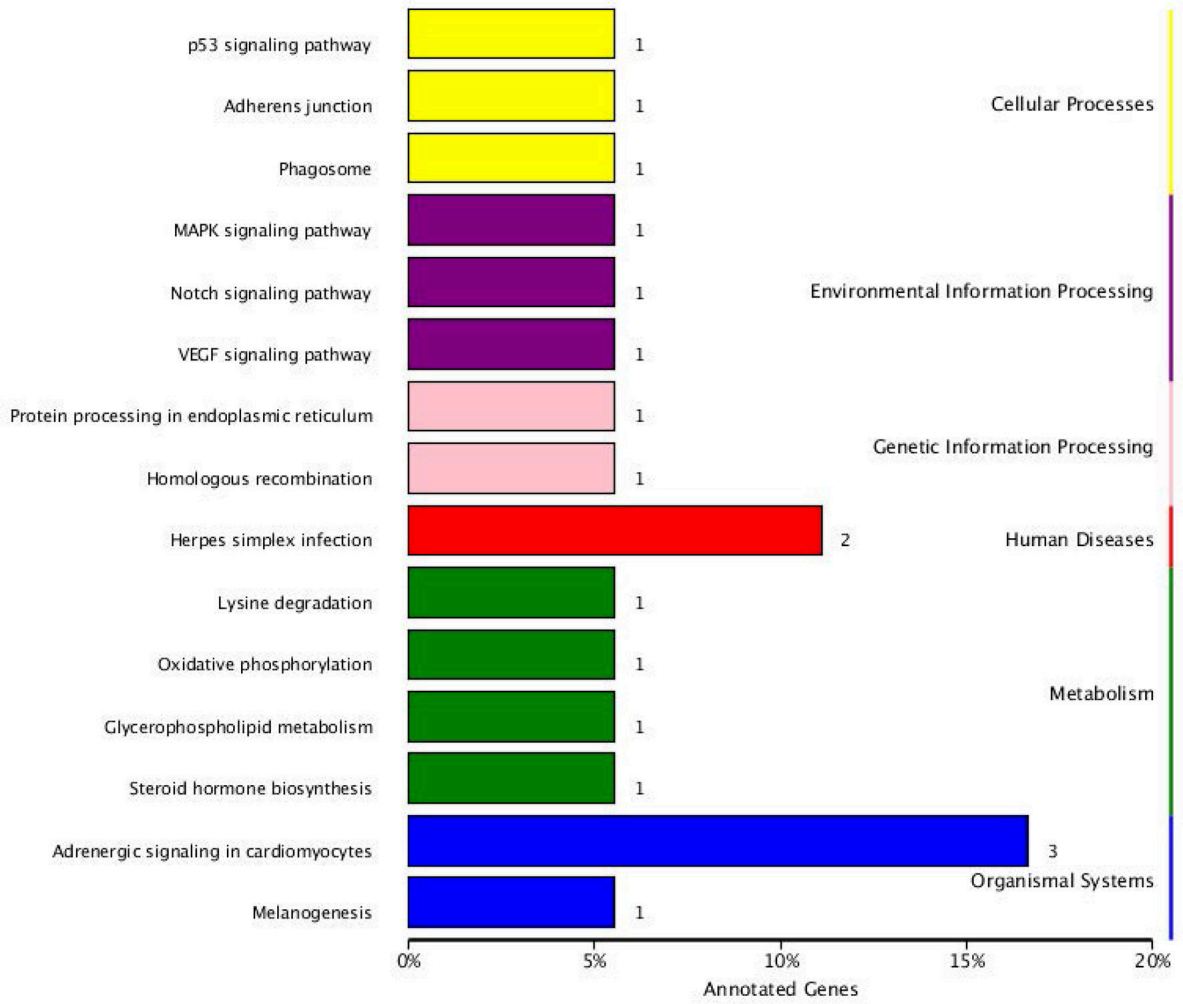
KEGG pathway annotation of circRNA parental genes.

The KEGG pathway analysis results showed that the parental genes of those SDE circRNAs were enriched in 15 pathways. Those enriched pathways were divided into three categories. (1) The metabolism-related including oxidative phosphorylation pathway, lysine degradation, glycerophospholipid metabolism, and steroid hormone biosynthesis. (2) The immune-related including p53 signaling pathway, MAPK signaling pathway, Notch signaling pathway, VEGF signaling pathway, Herpes simplex infection, and Adrenergic signaling in cardiomyocytes. (3) Other pathways including Adherens junction, Phagosome, Protein processing in the endoplasmic reticulum, Homologous recombination and Melanogenesis. The RYR2, TPM1, and TPM2 genes were included in the Adrenergic signaling in cardiomyocytes pathway. The CLOCK and USP7 genes were included in the Herpes simplex infection pathway (Figure 6).

The genes included in the GO terms of the immune system were retrieved from BioMart (http://asia.ensembl.org/biomart). Three immune-related genes, TXNDC9, JAG2, and NFATC2, were parental genes of SDE circRNAs NC_006088.4:132593578|132598993, NC_006092.4:52223625|52225021, NC_006092.4:52223625|52231585, NC_006107.4:13405274|13406817, NC_006107.4:13405274|13430298, NC_006107.4:13405277|13406817, NC_006107.4:13405791|13406817, NC_006107.4:13405791|13430298, NC_006107.4:13405791|13448229, NC_006107.4:13419173|13420895, and NC_006107.4:13444867|13448229 and related to cell redox homeostasis, multicellular organismal development, the B cell receptor signaling pathway, cellular response to DNA damage stimulus, positive regulation of transcription from RNA polymerase II promoter, positive regulation of B cell proliferation, cytokine production, and response to drug biological process (Table 4).

**Table 4:** Immune-related significantly differentially expressed circRNAs.

**gene_ID**	**circRNA_ID**	**Gene name**	**Regulation**	**GO-BP**	**KEGG pathway**
gene2149	Chr1:132593578-132598993	TXNDC9	Down	Cell redox homeostasis (GO:0045454)	
gene10321	Chr5:52223625-52225021	JAG2	Down	Multicellular organismal development (GO:0007275);	Notch signaling pathway
gene19025	Chr20:13405274-13406817	NFATC2	Up	B cell receptor signaling pathway (GO:0050853); Cell migration (GO:0016477); Cellular response to DNA damage stimulus (GO:0006974); Positive regulation of transcription from RNA polymerase II promoter (GO:0045944); Positive regulation of B cell proliferation (GO:0030890); Cytokine production (GO:0001816); Response to drug (GO:0042493);	VEGF signaling pathway

### Interaction Between circRNAs, miRNAs, and Genes

**Figure 7:**
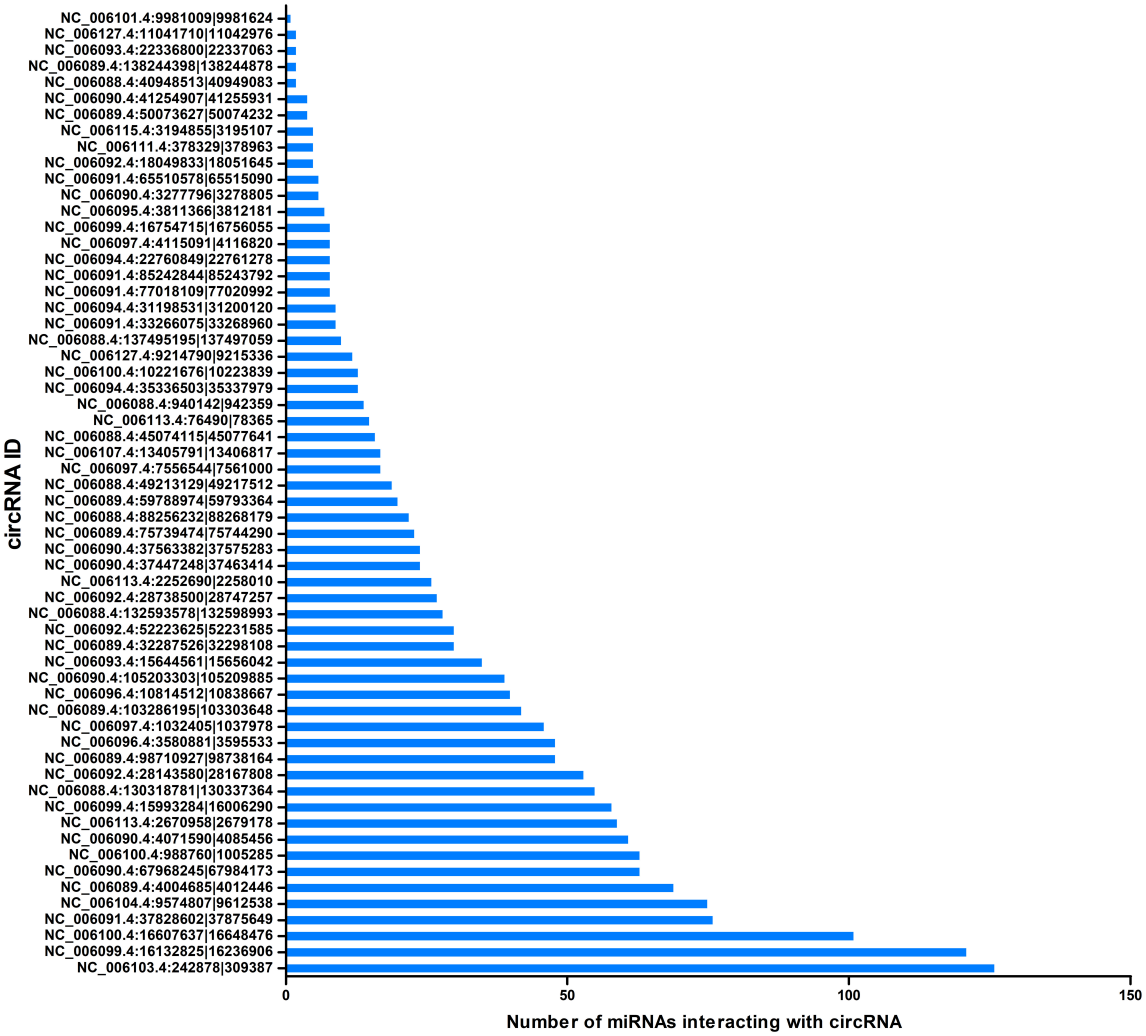
Correlation between miRNAs and circRNAs. The X-axis represents the number of miRNAs interacting with circRNA. The Y-axis represents the circRNA name.

**Figure 8:**
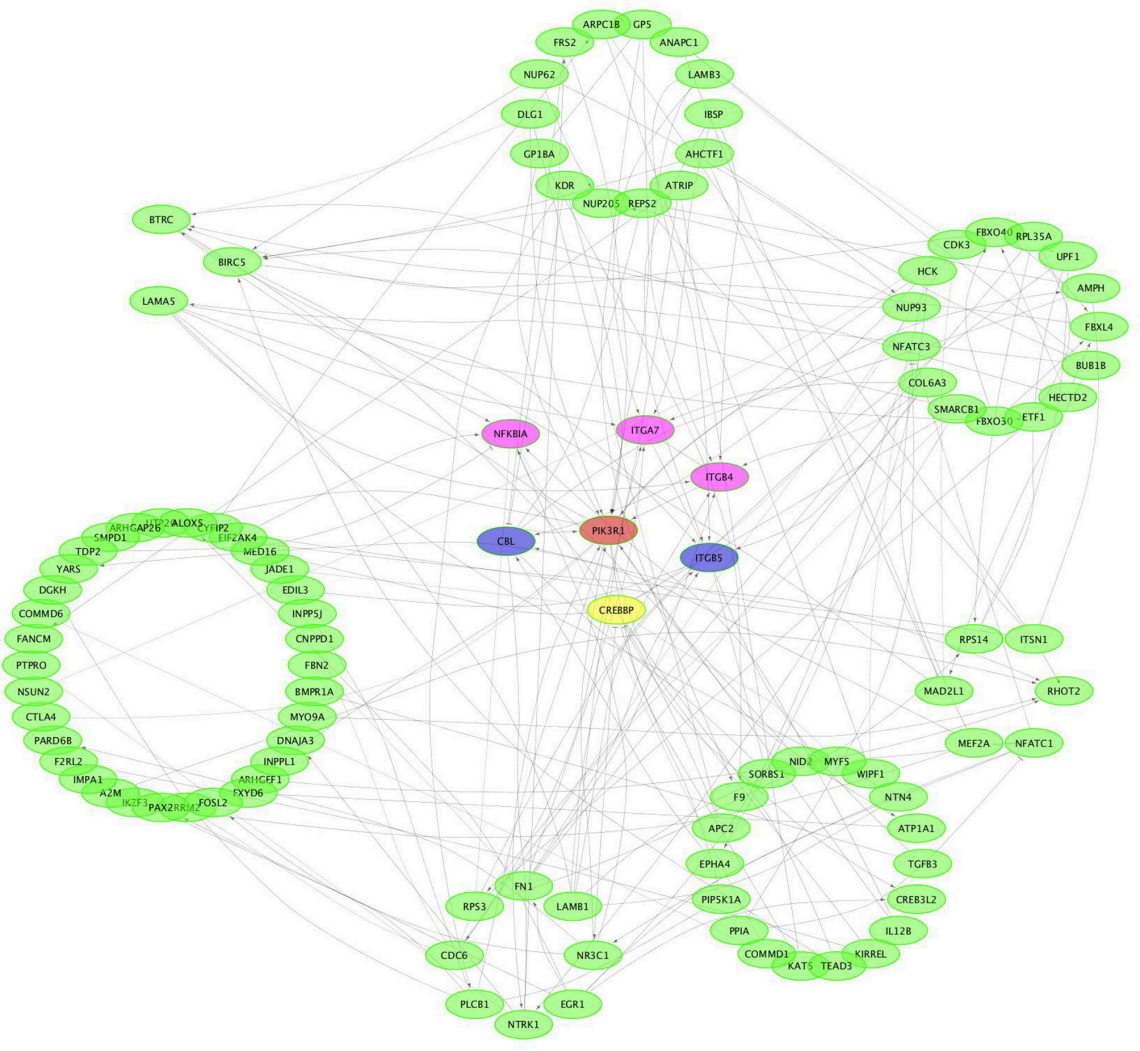
Protein-protein interaction network of potential genes targeted by gga-miR-34a-5p.

CircRNAs can act as miRNA sponges. The miRNAs interacting with the SDE circRNAs were predicted using miRanda. There were 1,787 interaction incidents identified between the 60 SDE circRNAs and 624 miRNAs (Supplementary File 3). The number of miRNAs interacting with different circRNAs was not even. CircRNA NC_006103.4:242878|309387, NC_006099.4:16132825|16236906, and NC_006100.4:16607637|16648476 interacted with more than 100 miRNAs. Ten circRNAs interacted with 50–100 miRNAs. Twenty-seven circRNAs interacted with 10–50 miRNAs. Twenty circRNAs interacted with < 10 miRNAs. CircRNA NC_006101.4:9981009|9981624 interacted with only one miRNA of gga-miR-1756a (Figure 7). One miRNA interacted with different circRNAs. Gga-miR-6545-3p, gga-miR-1696, gga-miR-1768, gga-miR-6553-5p, gga-miR-6573-5p, gga-miR-34a-5p, gga-miR-449c-5p, and gga-miR-6549-5p interacted with more than 10 circRNAs. One hundred and nineteen miRNAs interacted with 5–9 circRNAs. More than one third (212/624) miRNAs interacted with one circRNA. The immune function related gga-mir-34a-5p, located on Chr16, regulated 629 target genes. The interaction network of proteins encoded by those target genes was constructed through the Reactome FI network plug-in using Cytoscape 3.5.1. Seven proteins connected with more than 7 other proteins. PIK3R1 connected with 20 proteins, CBL and ITGB5 connected with 10 proteins, CREBBP connected with 9 proteins. NFKBIA, ITGAT, and ITGB4 connected with 8 proteins. Thirty-seven proteins connected with 4–7 other proteins, and 94 proteins connected with < 4 other proteins (Figure 8).

### Validation of circRNA Expression by qRT-PCR

Three circRNAs including two upregulated (NC_006113.4:2670958|2679178, NC_006096.4:10814512|10838667) and one downregulated (NC_006115.4:3194855|3195107) were randomly selected for validation by qRT-PCR. The expression of each of all the validated circRNAs detected by qRT-PCR was consistent with that detected by the sequencing in terms of the regulatory direction and significance. CircRNAs NC_006113.4:2670958|2679178 and NC_006096.4:10814512|10838667 were upregulated with a fold change of 27.60 and 5.95 by qRT-PCR, respectively. CircRNA NC_006115.4:3194855|3195107 was downregulated with a fold change of 3.13 (Table 5).

**Table 5:** Fold change of three differentially expressed circRNAs by qRT-PCR and NGS.

**CircRNA ID**	**qRT-PCR**	**NGS**
NC_006113.4:2670958|2679178	27.60^**^	2.80^*^
NC_006096.4:10814512|10838667	5.95^*^	Inf
NC_006115.4:3194855|3195107	−3.13^**^	−16.70^**^

* P < 0.05;

** *P < 0.01*.

## Discussion

CircRNAs are recently discovered noncoding RNAs and have attracted significant attention (34). In the current study, circRNAs related to SE inoculation in chickens have been revealed through next-generation sequencing. A close number of upregulated and downregulated circRNAs were identified in several circRNAs profiling studies (18, 34). Similar results were found in the current study since the number of significantly upregulated and downregulated circRNAs was very close (30 vs. 32) in the chicken cecum following SE inoculation (Supplementary File 2). It has been reported that the number of upregulated and downregulated genes (115 vs. 37) was significantly different; however, the number of upregulated and downregulated miRNAs (22 vs. 15) following SE inoculation was similar (35, 36). When a persistent immunity to SE inoculation is established, the host immune signaling pathways can be modulated (37). Many immune-related functional terms and pathways have been found following Salmonella inoculation in the chicken (38–40). In the current study, only two immune-related functional terms (response to stimulus and immune system process) were enriched (Figure 5). The regulatory roles played by the genes, miRNAs, and circRNAs in the response to SE inoculation in the chicken were different. It has been reported that circadian rhythms can influence mammal immune response through regulating the blood circulation during diurnal sleeping/waking cycles (41, 42). Domestic pigs exhibit diurnal rhythms in peripheral blood immune cell numbers (43). It has been reported that the circadian rhythm was significantly enriched in the chicken cecum following *Campylobacter jejuni* (*C. jejuni*) infection (44). In the current study, the circadian rhythm-associated circRNAs were significantly triggered by SE inoculation. A different circadian rhythm regulation mechanism could exist in response to *C. jejuni* compared to that of SE inoculation.

Metabolism is important to facilitate the requirements for energy and biosynthesis and directly regulate immune cell functions. A bacterial infection competes for nutrients with immune cells (45). The differentially expressed circRNAs were enriched in metabolism-related KEGG pathways including the oxidative phosphorylation pathway, lysine degradation, glycerophospholipid metabolism, and steroid hormone biosynthesis following SE inoculation (Figure 6). Upon encountering an antigen, lymphocytes switch into the specific effector state with metabolism changes (46). Those metabolic changes are required not only for lymphocyte plasticity but also for T cell fate (47). Salmonella infection induces rapid and robust T-cell activation in mammalians (48). Changes in metabolic activity have been shown to intimately support T cell differentiation and effector functions (49). The oxidative phosphorylation pathway is the significant energy generating system in animals and it is highly conserved in insects and vertebrates (50). Naïve T cells rely on oxidative phosphorylation to maintain the energy demand; in contrast, activated T cells engage in aerobic glycolysis consuming massive amounts of glucose (51). Oxygen and reactive oxygen species metabolism were enriched following SE infection in chickens (35). The oxidative phosphorylation pathway is a prime candidate for cytonuclear genomic incompatibilities, and ATPases are composed of subunits from both the nuclear and mitochondrial genomes (52, 53). It has been reported that supplementation with lysine-yielding *Bacillus subtilis* in the diet increased intestinal immune response in Linwu ducks (54). It has been reported the balance between metabolism and the immune system contributes to the response to SE inoculation in chickens (36, 55).

CircRNAs were identified as efficient microRNA (miRNA) sponges (16, 17). Many studies have indicated that circRNAs regulate the function of miRNAs acting as competing endogenous RNAs (ceRNAs) (16, 56, 57). miR-143 and miR-26 are differentially expressed in whole blood after Salmonella inoculation in pigs (58). Gga-miR-101-3p and gga-miR-155 were identified as candidates potentially associated with SE infection in the chicken (59). It has been reported that gga-miR-125b-5p, gga-miR-34a-5p, gga-miR-1416-5p, and gga-miR-1662 play an important role in SE infection (55). miRNAs buffer and alter the variance of relatively low expressed genes in response to Salmonella infection in pigs (60). In the current study, gga-miR-125b-5p and gga-miR-34a-5p interacted with 3 and 10 circRNAs, respectively (Supplementary File 3). Proteins encoded by gga-miR-34a-5p-mediated genes had close interaction (Figure 8). The results showed that circRNA may have interacted with miRNA in the response to SE inoculation in chickens.

In poultry, circadian rhythms are generated from the transcription/translation-based oscillatory loop including Per2, Per3, CLOCK, and Bmal1 (61–63). Circadian disruptions have been well-documented in adverse effects on human health through influencing lipid and glucose homeostasis, inflammation, and cardiovascular functions (64). Studies have shown that a set of cytokines, IL-6, IL- 1β, IL-18, IL-2, TGF-β4, K60, and IL-8, and circadian clock genes (cry1/2, per2/3, Bmal1/2, and CLOCK) have a 24-h periodic expression pattern in response to bacterial colonization (65, 66). Furthermore, the CLOCK gene was involved in the herpes simplex infection pathway and related to human disease. The CLOCK gene was significantly changed post *C. jejuni* inoculation (67). In the current study, the SDE circRNA NC_006091.4:65510578|65515090 originated from the CLOCK gene was significantly upregulated (Supplementary File 2) and could play an important role in response to SE inoculation.

Forkhead box proteins (FOXP) are part of a large transcription factor family with diverse functions in development, metabolism, organogenesis, and cancer (68). FOXP1, a member of the “FOXP” subfamily, is an essential transcriptional regulator for B lymphopoiesis (69, 70) and the generation of quiescent naïve T cells during thymocyte development (71). Disruption of FOXP1 leads to cognitive dysfunction including intellectual disability and autism spectrum disorder together with language impairment (72). A gene could be spliced into one or more circRNAs (73). circ-SHKBP1 regulated the angiogenesis of glioma-exposed endothelial cells through the miR-544a/FOXP1 and miR-379/FOXP2 Pathways (74). The level of miR-152 and FOXP1 was inversely correlated in grade 3 and 4 ovarian tumor tissues (75). Two circRNAs NC_006099.4:16132825|16236906 and NC_006099.4:15993284|16006290 originated from FOXP1 were significantly expressed with reverse regulatory direction (Supplementary File 2). Those two circRNAs could regulate the response to SE inoculation through FOXP1 and miRNAs in the chicken. The mechanism of interaction between circRNAs and FOXP1 in the response to SE inoculation in the chicken needs to be further warranted.

## Conclusions

In conclusion, circRNAs were involved in the response to SE inoculation in the chicken. CircRNAs associated with the immune system process, the rhythmic process and metabolic process contribute to the response to SE inoculation. CircRNAs NC_006091.4:65510578|65515090, NC_006099.4:16132825|16236906, and NC_006099.4:15993284|16006290 play critical roles in the response to SE inoculation. The findings herein will provide fundamental information on the mechanism of circRNAs regulating the response to SE inoculation in the chicken.

## Ethics Statement

All animal procedures were approved by Shandong Agricultural University Animal Care and Use Committee.

## Author Contributions

LZ and LLu performed the experiments, analyzed the data, and drafted the manuscript. LLn performed the experiments and collected samples. HT and XF provided chickens and helped to analyze the data. HL reviewed the manuscript. XL designed the experiment and reviewed the manuscript.

### Conflict of Interest Statement

The authors declare that the research was conducted in the absence of any commercial or financial relationships that could be construed as a potential conflict of interest.

## Supplementary Material

Supplementary File 1 The coverage of circRNAs in each chromosome.

Supplementary File 2 Information of the significantly differentially expressed circRNAs.

Supplementary File 3 Correlation between circRNAs and miRNAs.
